# Detection of Hydroxyl Radicals Using Cerium Oxide/Graphene Oxide Composite on Prussian Blue

**DOI:** 10.3390/nano10061136

**Published:** 2020-06-09

**Authors:** Surachet Duanghathaipornsuk, Sushil Kanel, Emily F. Haushalter, Jessica E. Ruetz, Dong-Shik Kim

**Affiliations:** 1 Department of Chemical Engineering, 2801 W. Bancroft St., University of Toledo, Toledo, OH 43606, USA; Surachet.Duanghathaipornsuk@rockets.utoledo.edu (S.D.); Emily.Haushalter@rockets.utoledo.edu (E.F.H.); Jessica.Ruetz@rockets.utoledo.edu (J.E.R.); 2 Office of Research & Sponsored Programs, Air Force Institute of Technology, 2950 Hobson Way, Wright-Patterson AFB, OH 45433, USA; Sushil.Kanel@afit.edu

**Keywords:** hydroxyl radicals, cerium oxide, Prussian blue, graphene oxide, composite sensor, cyclic voltammetry, electrochemical method, Prussian blue degradation

## Abstract

A composite sensor consisting of two separate inorganic layers of Prussian blue (PB) and a composite of cerium oxide nanoparticles (CeNPs) and graphene oxide (GO), is tested with •OH radicals. The signals from the interaction between the composite layers and •OH radicals are characterized using cyclic voltammetry (CV). The degradation of PB in the presence of H_2_O_2_ and •OH radicals is observed and its impact on the sensor efficiency is investigated. The results show that the composite sensor differentiates between the solutions with and without •OH radicals by the increase of electrochemical redox current in the presence of •OH radicals. The redox response shows a linear relation with the concentration of •OH radicals where the limit of detection, LOD, is found at 60 µM (100 µM without the PB layer). When additional composite layers are applied on the composite sensor to prevent the degradation of PB layer, the PB layer is still observed to be degraded. Furthermore, the sensor conductivity is found to decrease with the additional layers of composite. Although the CeNP/GO/PB composite sensor demonstrates high sensitivity with •OH radicals at low concentrations, it can only be used once due to the degradation of PB.

## 1. Introduction

Hydroxyl radicals (•OH radicals) are one of the most reactive free radicals among reactive oxygen species (ROS). In a human body, •OH radicals are produced as a by-product of cellular respiration primarily in the mitochondria [1,2], the oxidation burst in phagocytic cells [3,4], and enzyme reactions [5,6] for various cellular functions such as restoration of damaged DNA [7], activating vital proteins [8,9], signaling pathways [10], and responding to external impacts [11]. The imbalance between production and elimination of •OH radicals occurs due to the overproduction of ROS or oxidants beyond the capability of cells to facilitate an effective antioxidant response [12,13]. The excess of •OH radicals could develop the oxidative stress condition in a human body leading to interference of the normal function of cells [14] and damage of cellular components including DNAs [15,16], and lipids [17,18]. Acceleration of aging, cancer, cardiovascular diseases, and neurodegenerative diseases, such as Alzheimer’s disease and Parkinson’s disease, are a few examples of the negative impacts from the oxidative stress [19,20,21]. The detection of •OH radicals, as a biomarker, therefore, is a crucial step in the diagnosis of those severe diseases at initial stages.

Unfortunately, it is exceptionally challenging to detect •OH radicals, and other ROS in general, because they have extremely short lifetimes. In addition, due to their high chemical reactivity, they can easily destroy or disrupt the sensing elements of detection devices preventing them from generating and transducing trustful signals [22,23,24]. A sensor technology enabling real-time detection of •OH radicals with high sensitivity and selectivity would be beneficial to medical diagnoses for such diseases at early stages [25,26,27].

Several direct and indirect detection methods have been developed for •OH radicals. Electron paramagnetic resonance spectroscopy (EPR) coupled with spin trapping is a common method for direct detection of •OH radicals [28,29]. This method requires high and constant concentrations of •OH radicals to get a reliable result, which is often hardly achieved due to the short lifetime and high reactivity of •OH radicals. An aliquot of sample should be taken from a source of •OH radicals—for example—via a biopsy, and analyzed with EPR, and in this procedure, contents of •OH radicals, may change significantly. Most of indirect methods are using a chemical derivatization with a separation technique including high-performance liquid chromatography (HPLC) coupled with either UV spectroscopy [30,31], mass spectrometry [32,33], fluorescence method [34,35], or electrochemical detection (ED) [36,37]. For instance, the HPLC-ED identifies and also quantifies •OH radicals by measuring hydroxylation products from the reaction between •OH radicals and aromatic molecules [38]. Indirect methods require two-step procedure—reaction and separation—and just like the direct methods, they also have to undergo the sampling procedure, during which the accuracy of the measurement reduces.

In the current study, a real-time composite sensor for detecting •OH radicals has been developed by depositing two separate inorganic layers on a screen printed glassy carbon electrode (GCE). A GCE is selected because of its low cost and good compatibility with most materials. The first layer applied to a working GCE is Prussian blue (PB) as an electrocatalyst to mediate redox reactions. It is hypothesized that PB can increase the conductivity and sensitivity of the composite sensor at low concentrations of •OH radicals. The sensitivity of sensor is an important factor in the detection of free radicals because it is necessary to be able to analyze even a small abnormal increase of •OH radicals at an onset of oxidative stress-related diseases. The first layer of PB is formed on a GCE by the electrochemical deposition, in which PB is deposited on the surface of electrode by electrostatic self-assembly [39]. The second layer of the sensor is a composite mixture of CeO_2_ nanoparticles (CeNPs) with graphene oxide (GO), i.e., a CeNP/GO composite. CeNPs are employed as a sensing element with the active sites for the reaction with •OH radicals [40,41]. The detection of •OH radicals occurs as Ce^3+^ on a CeNP reacts with •OH radicals and turns into Ce^4+^. In the oxidation-reduction cycle, a CeNP alternates between the oxidation states of Ce^4+^ and Ce^3+^ [42], and therefore, the reaction with •OH radicals can be monitored by transmitting the electrical signals [40,43,44].

It is hypothesized that the use of CeNP provides the sensor with the desired selectivity towards •OH radicals. Also, the reversible reaction of CeNPs with •OH radicals enables the sensor to be reusable and capable of real-time detection. As for GO, it is hypothesized that GO enhances the electron transfer rate on the sensor surface when combined with CeNPs due to its intrinsic properties such as large surface area and high conductivity [45]. Electrochemical reduction is implemented to fully exploit the intrinsic conductivity of GO. Several groups have proved that the conductivity of GO significantly increased after being treated with electrochemical reduction [46]. To characterize the presumed sensitivity, reusability, and capability of real-time detection, cyclic voltammetry (CV) was used. To the best of our knowledge, this is a first real-time electrochemical sensor with the integration of PB and CeNPs for the detection of •OH radicals in an aqueous system.

## 2. Materials and Methods

### 2.1. Materials

Cerium(IV) oxide nanopowder, graphene oxide, potassium hexacyanoferrate(II) trihydrate (98.5–102.0%), potassium hexacyanoferrate(III) (>99%), iron(III) chloride (97%), iron(II) sulfate heptahydrate (≥99%), potassium chloride (≥99%), and hydrogen peroxide (30% w/w) were obtained from Sigma-Aldrich (St. Louis, MO, USA). Screen printed carbon electrodes (GCE) (Pine Instruments, Grove City, PA, USA) were used as a sensor base with a 2 mm-working electrode. The counter electrode and the reference electrode were carbon and Ag/AgCl, respectively. The sizes of CeNPs and the presence of CeNPs in the composites were investigated by using a scanning transmission electron microscope, STEM Hitachi HD-2300A (Tokyo, Japan). The bare and modified electrode were recorded using a field emission scanning electron microscopy, SEM Hitachi S-4800 (Tokyo, Japan). The composition of the CeNP/GO composite was confirmed by using a Rigaku Ultima III X-ray diffractometer with small angle X-ray scattering (SAXS). Cyclic voltammetry (CV) was performed using a Gamry Reference 600 potentiostat (Gamry Instruments, Warminster, PA, USA).

### 2.2. Synthesis of CeNP/GO Composite

Both CeNPs and GO (50 mg each) were added into 100 mL of deionized water. The mixing solution was then placed into an ultra-sonication bath for one hour. Following sonication, the mixing solution was stirred for two hours to form a composite. The homogeneous mixing solution was then transferred to a centrifuge tube and centrifuged at 12,000 rpm for 30 min to receive the precipitated solid from the liquid portion of the mixing solution. The composite sample was then collected and dried at 60 °C for 12 h [47]. Once dried, the solid was grounded to a fine powder and kept in a desiccator at room temperature. The final CeNP/GO composite was confirmed by SAXS and STEM.

### 2.3. Deposition of PB on a GCE

The deposition of PB on a GCE was reported in previous literatures [48,49]. Briefly, before any PB was immobilized on a working electrode, a GCE was cleaned with 0.1 N sulfuric acid using CV to eliminate impurities on the surface of electrode. After that, two solutions were prepared to deposit PB on the working electrode. The first solution was made of 2 mM potassium ferricyanide, K_3_[Fe(CN)_6_]; 0.1 M potassium chloride, KCl; and 0.1 M hydrochloric acid, HCl. The second solution contained 2 mM iron(III) chloride, FeCl_3_, at pH of 2. The both solutions were mixed with an equal volume of 40 mL, and the mixture was referred as a PB growing solution. CV was performed in the potential range from 0.3 to 0.8 V with 40 mV/s of scan rate for 15 cycles. After the color of working electrode turned into light blue, PB was stabilized by executing CV in the activating solution of 0.1 M KCl and 0.1 M HCl with the potential range of −0.05 V to 0.35 V at a scan rate of 40 mV/s for 10 cycles. Once the activation was completed, the electrode was dried at 80 °C for one hour. The PB deposition was assured by performing CV in the range of −0.8 V to 0.8 V with 100 mV/s in a 0.1 M phosphate buffer solution (PBS, pH 7.2) containing 5 mM of [Fe(CN)_6_]^3−/4−^ and 0.1 M KCl.

### 2.4. Preparation of the CeNP/GO Composite on the PB-Modified GCE

10 mg of the CeNP/GO composite powder were suspended in 10 mL of deionized water. The solution was then sonicated for one hour to obtain a homogenous solution. The CeNP/GO composite solution was applied to the PB-modified glassy carbon working electrode by delivering 10 μL with a pipette and dried in an oven at 60 °C for one hour. After drying, the CeNP/GO composite was reduced by CV through electrochemical reduction with the potential range from 1.7 to −1.7 V at 40 mV/s for 12 cycles to improve the overall composite conductivity [45]. Then, the CeNP/GO composite layer on top of the PB layer was rinsed with deionized water and dried again under nitrogen gas. CV was used to confirm the presence of composite layer on top of the PB-modified glassy carbon working electrode with the potential range between −0.8 V to 0.8 V at a scan rate of 100 mV/s in the same CV solution used in 2.3.

### 2.5. Detection of •OH Radicals by CeNP/GO/PB on a GCE

To test the composite sensor, •OH radicals were generated using the Fenton reaction. 10 mM of H_2_O_2_ solution was mixed with 10 mM solution of FeSO_4_·7H_2_O with an equal volume to perform the Fenton reaction. The H_2_O_2_ solution was covered with aluminum foil to prevent the oxidation from UV light exposure for the duration of the experiment. CV was implemented to test the sensor during the Fenton reaction. The first cycle in CV was run in the H_2_O_2_ solution. After that, the test was paused and an equal volume of the FeSO_4_·7H_2_O solution was added to the H_2_O_2_ solution to begin the Fenton reaction. CV was continuously used to detect the current change of the sensor during the Fenton reaction with the potential range of −0.6–0.4 V at 100 mV/s. After the Fenton reaction terminated within 15 min, the sensor was transferred to the same CV solution used in 2.3 and CV was run to check for the degradation of PB and composite layers on the surface of electrode.

**Figure 1:**
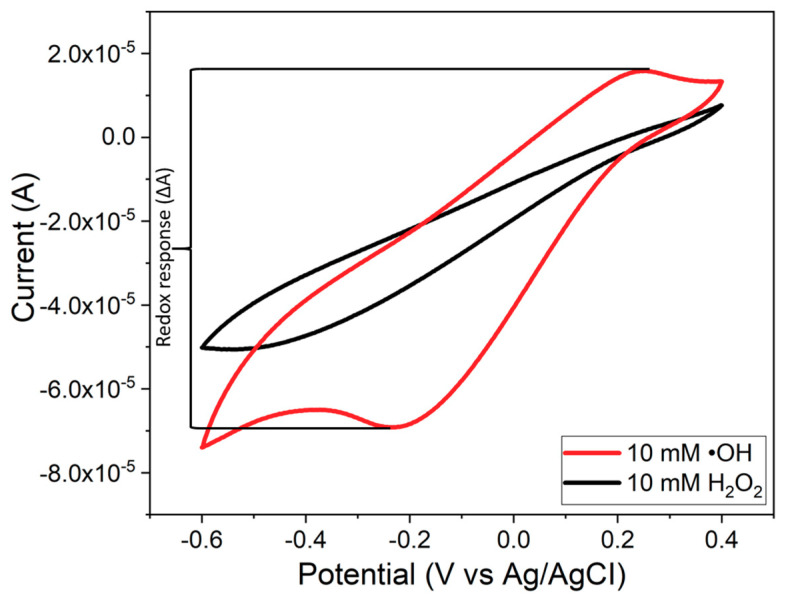
Calculation of the redox response (ΔA) to hydroxyl radicals.

**Figure 2:**
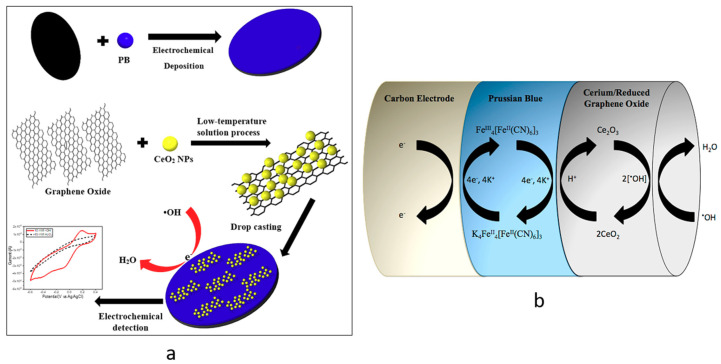
(**a**) Schematic of the construction and test processes for the composite sensor, (**b**) the mechanism of detection of hydroxyl radicals in the sensor design.

After testing, the sensors were washed with deionized water and dried under nitrogen gas for next tests. The same test procedure was repeated for a sensor multiple times to investigate the reusability of sensor. Both the reduction and oxidation responses (i.e., redox responses) in the cyclic voltammogram were used to calculate the redox response (*ΔA*) of the sensor due to the redox reaction between the CeNP/GO composite and •OH radicals. The redox response in terms of the current change (*ΔA*) was calculated using the procedure described in Figure 1, in which *ΔA* is taken from the difference between the currents at the oxidation and reduction peaks. The CV curve for H_2_O_2_ shows no significant redox peaks, which proves that there is no considerable redox reaction between the CeNP/GO modified electrode and H_2_O_2_. Figure 2a summarizes the synthesis of the CeNP/GO/PB modified electrode and the detection of •OH radicals in the Fenton solution. The design concept of the sensor is also shown in Figure 2b.

## 3. Results and Discussion

### 3.1. Synthesis and Characterization of the CeNP/GO Composite

**Figure 3:**
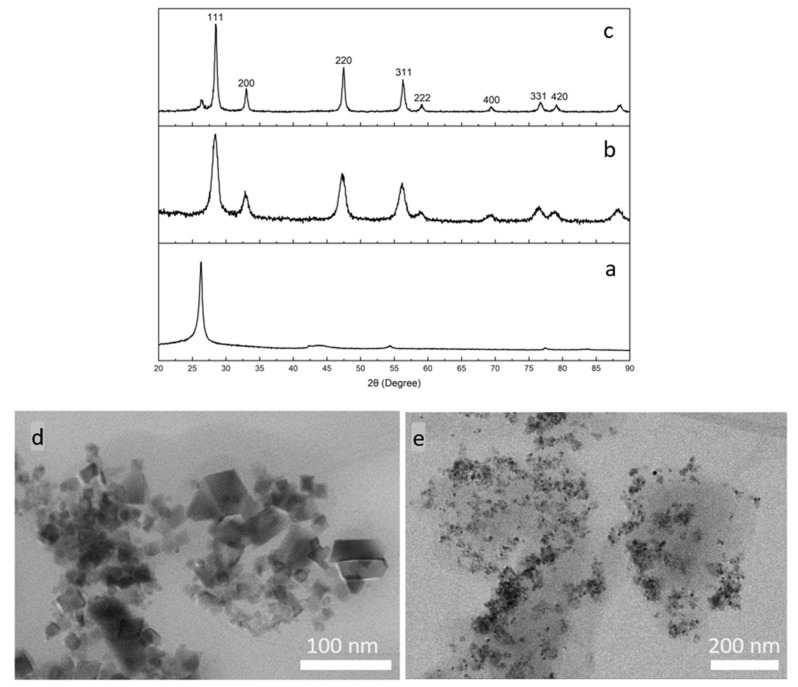
XRD patterns of (**a**) GO, (**b**) CeNPs, and (**c**) CeNP/GO composite; and STEM images of (**d**) CeNPs and (**e**) CeNP/GO composite.

The composite was synthesized by a low-temperature solution process. The XRD patterns of GO, CeNPs, and the CeNP/GO composite are showed in Figure 3a–c, respectively. Figure 3b shows the crystalline structure of CeNPs with the refractive indexes at 28.4° (111), 32.9° (200), 47.3° (220), 56.1° (311), 58.8° (222), 69.3° (400), 76.5° (331), and 78.9° (420), which consistent with the standard cubic structure of CeO_2_ (JCPDS 65-2975) [50,51]. As for the XRD pattern of the CeNP/GO composite, Figure 3c demonstrates the crystalline structure of CeNPs which confirms the presence of CeNPs in the composite. It is worth mentioning that the refractive index of the CeNP/GO composite spikes with a sharper peak in comparison to that of CeNPs, which is attributed to a highly ordered CeNP crystallinity in the composite. On the other hand, it is observed that the characteristic XRD pattern of GO around 25° significantly reduces in the CeNP/GO composite, which is thought to be due to the disorder of stacking of graphene oxide sheets in the composite.

The morphologies of CeNPs and the CeNP/GO composite were investigated using STEM. Figure 3d,e show the bright field TEM images of CeNPs and CeNP/GO composite, respectively. In Figure 3d, CeNPs have an average size from 15 nm to 60 nm with a consistent cubic shape. For the CeNP/GO composite, which is exhibited in Figure 3e, CeNPs are homogeneously dispersed all over the GO sheets. Thus, it is confirmed that the low-temperature solution process can be successfully used to prepare the CeNP/GO composite.

### 3.2. Characterization of the PB Layer Deposited on a GCE

**Figure 4:**
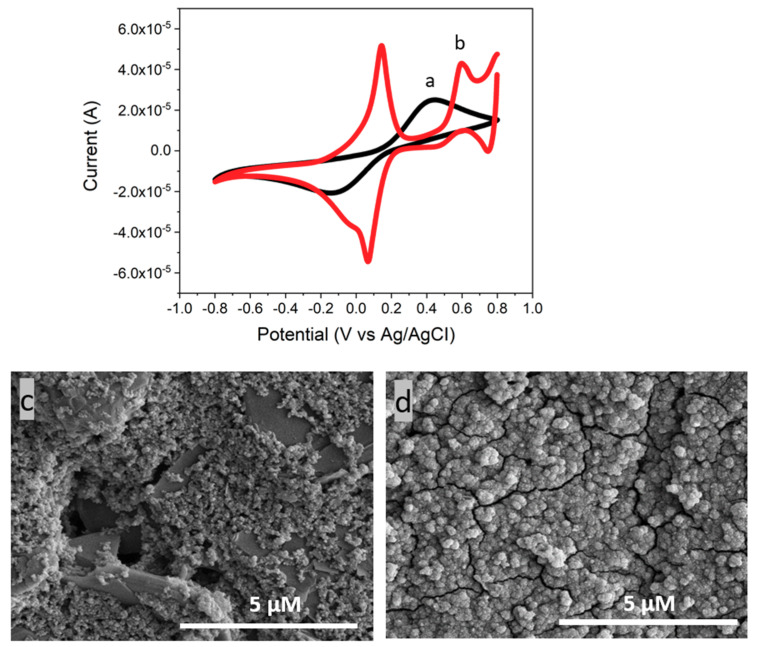
CV results of (**a**) a bare screen printed carbon electrode (GCE), and (**b**) PB deposited on a GCE in a 0.1 M phosphate buffer solution (PBS, pH7.2) containing 5 mM of [Fe(CN)_6_]^3−/4−^ and 0.1 M KCl with 100 mV/s of scan rate. SEM images of (**c**) a bare GCE and (**d**) PB deposited on a GCE.

The CV results for a bare GCE and the PB modified GCE are shown in Figure 4a,b. Once the electrochemical deposition was performed, two distinct redox peaks appear in the cyclic voltammogram for the PB modified electrode as shown in Figure 4b. These two redox peaks, which are found at 0.1 V and 0.6 V, represent the reduced form (Prussian white) and the oxidized form (Berlin green) of PB, respectively. Furthermore, the PB modified GCE shows a higher conductivity in comparison to the bare GCE. The increase of sensor conductivity is explained with an intrinsic characteristic of PB as an electrocatalyst. PB is well-known for its redox catalysis that increases a rate of electron transfer in a redox reaction between an electrode surface and electrolyte in a solution [52,53]. The addition of a PB layer on the electrode surface as an interlayer between the electrode and the CeNP/GO composite layer can facilitate the electron transfer resulting in an increase in the sensor conductivity [54,55].

Additionally, SEM was used to investigate the morphologies of the deposited PB layer on a GCE. Figure 4c,d are SEM images of a bare GCE and the PB modified GCE, respectively. Figure 4c shows an uneven surface of GCE. After the electrochemical deposition of PB, a homogenous PB layer across the electrode surface was formed as shown in Figure 4d. Thus, it is confirmed that, from the CV and SEM results, the electrochemical deposition is successfully used to deposit a PB layer on the electrode surface.

### 3.3. Characterization of CeNP/GO/PB on a GCE

**Figure 5:**
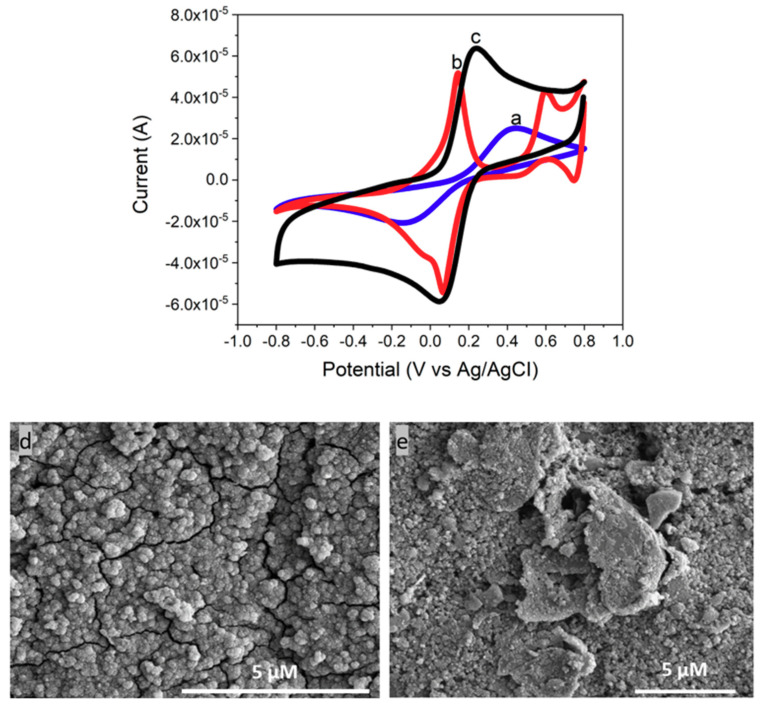
CV responses of (**a**) a bare screen printed carbon electrode (GCE), (**b**) PB deposited on a GCE, and (**c**) CeNP/GO/PB modified GCE in a 0.1 M phosphate buffer solution (PBS), pH 7.2 containing 5 mM of [Fe(CN)_6_]^3−/4−^ and 0.1 M KCl with 100 mV/s of scan rate. SEM images of (**d**) PB deposited on a GCE and (**e**) CeNP/GO/PB modified GCE.

The composite layer was deposited on an electrode surface using the drop casting method. The chemisorption interaction is responsible for the attachment of the CeNP/GO composite with the PB modified electrode. CV was employed to verify the deposition of CeNP/GO composite on top of the PB modified electrode. As shown in Figure 5, two redox peaks of PB turn into one redox peak of the CeNP/GO composite modified sensor. Furthermore, the electrode conductivity increases after applying the CeNP/GO composite layer on top of the PB modified electrode, which is attributed to the highly conductive GO in the composite. The potential change (*ΔE_p_*) of the oxidation and reduction peaks also decreases for the composite modified sensor. The shift of redox peaks either to positive or negative potential indicates the reversibility of redox reaction at the electrode surface as a peak-to-peak separation (*ΔE_p_*). The *ΔE_p_*’s of a bare and the composite on the PB modified electrode are 980 mV and 170 mV, respectively. This result indicates that PB in the composite tremendously enhances the electron transfer for the redox reaction at the surface of electrode, which results in the significant reduction of *ΔE_p_*. Furthermore, SEM images were used to confirm the presence of CeNP/GO composite layer on top of the PB modified electrode. As demonstrated in Figure 5d,e, the surface morphology of PB modified GCE is completely different from the image taken after depositing CeNP/GO composite on the PB layer. Figure 5e shows the homogeneous dispersion of CeNP/GO composite on top of the PB modified GCE. Therefore, it is concluded that the CeNP/GO composite layer was successfully deposited on the PB modified electrode, and it showed a higher conductivity and required a lower potential to operate than the bare and PB modified electrodes.

### 3.4. Electrochemical Reduction of the CeNP/GO Composite

**Figure 6:**
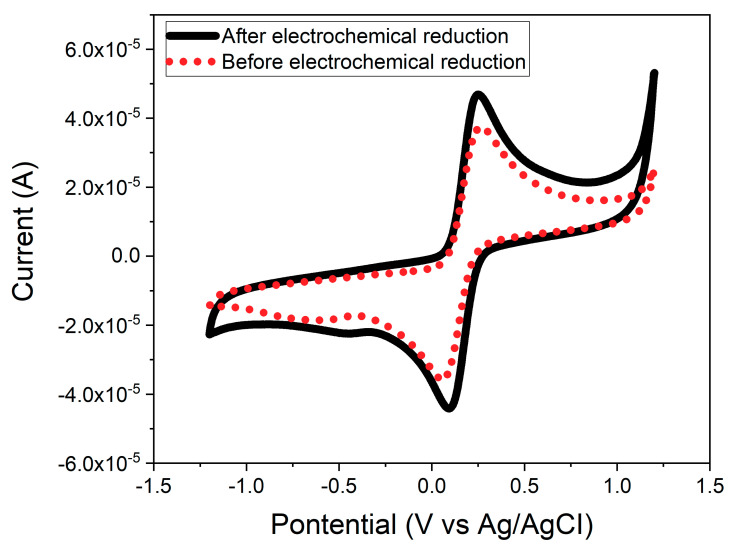
CV responses of the CeNP/GO composite before and after electrochemical reduction in a 0.1 M phosphate buffer solution (PBS, pH 7.2) containing 5 mM of [Fe(CN)_6_]^3−/4−^ and 0.1 M KCl with 100 mV/s of scan rate.

As mentioned earlier, electrochemical reduction can improve the intrinsic conductivity of GO. Figure 6 shows the cyclic voltammogram for the CeNP/GO composite modified electrode before and after the electrochemical reduction step. It is found that, the conductivity of CeNP/GO composite modified electrode significantly increases after treatment with electrochemical reduction. The increase in the conductivity of the CeNP/GO composite modified electrode is due to the elimination of oxygen groups on GO by electrochemical reduction.

### 3.5. Tests for •OH Radical Detection

#### 3.5.1. CV for •OH Radical Detection

**Figure 7:**
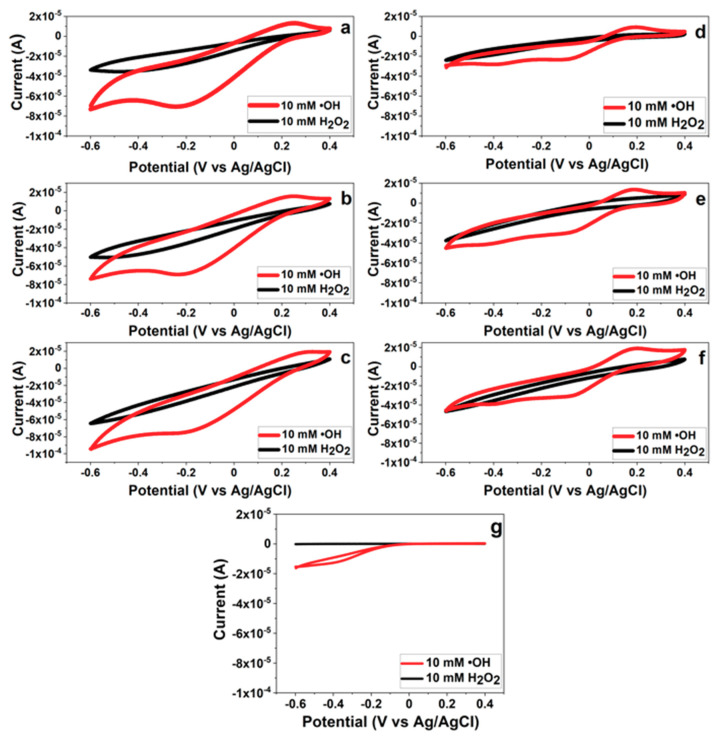
CV responses of (**a**,**d**) the single layer of CeNP/GO composite with and without the PB modification, (**b**,**e**) double layers of CeNP/GO composite with and without the PB modification, (**c**,**f**) triple layers of CeNP/GO composite with and without the PB modification, and (**g**) a bare screen printed carbon electrode (GCE) in the solution contains 10 mM of H_2_O_2_ and •OH radicals with the potential range from −0.6 V to 0.4 V, 100 mV/s.

As mention before, a CeNP has the dual oxidation states as Ce^3+^ and Ce^4+^ on the surface of particle. Several works have verified that the Ce^3+^ oxidation state on the surface of CeNP is responsible for the oxidation reaction with high selectivity toward •OH radicals [40,41]. Our hypothesis is that CeNPs possessing the Ce^3+^ oxidation state can be used as a sensing element for •OH radicals via the oxidation reaction. Figure 7 shows the cyclic voltammograms of three different layers of the CeNP/GO composite sensor with (7a, b, and c) and without the PB deposition (7d, e, and f) in the presence of H_2_O_2_ and •OH radicals. Regardless of the PB layer and additional composite layer(s), the CeNP/GO composite sensor shows the increase of oxidation current peak around 0.2 V in the presence of •OH radicals; in contrast, there is no oxidation current peak from the bare electrode. The composite shows greater reactivity with •OH than with H_2_O_2_ as Figure 7a shows, for example, that the redox response (*∆A*) for •OH radicals is 87 ± 6.2 μA while the *∆A* for H_2_O_2_ is 37 ± 0.5 μA. Therefore, it proves our hypothesis that CeNPs can be used as a sensing element and the Ce^3+^oxidation state on the surface of CeNP is the reactive site for •OH radicals.

**Figure 8:**
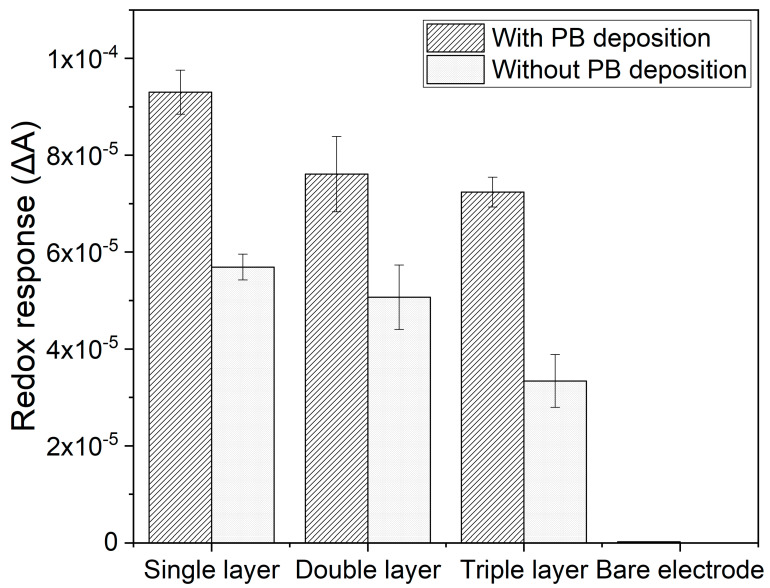
Redox responses (*∆A*) of the single, double, and triple layers of composite-modified electrodes with and without the PB deposition with 10 mM of •OH.

The CeNP/GO composite was catalyzed with PB to improve the conductivity and sensitivity of the sensor with low detection limits. The redox response (*∆A*) of three different layers of the composite with and without PB to •OH radicals is presented in Figure 8. As expected, the PB modified composite sensor delivers a significant increase in the *∆A* to •OH radicals compared to the composite sensor without the PB modification. Therefore, this experimental result confirms that the PB layer can be used as an electrocatalyst in this composite sensor configuration.

It was found, however, that the PB layer degraded after contacting with H_2_O_2_ or •OH radicals. In order to prevent the degradation of PB layer, additional layers of the CeNP/GO composite were deposited on top of the PB layer. It was thought that the extra layers of the composite deposited on top of the PB layer would prevent the degradation of PB layer. As shown in Figure 8, the addition of composite layers is found to reduce *∆A* of the composite sensor in the presence of •OH radicals. This could be due to the additional layer(s) enhances agglomeration of the nanoparticles that results in the reduction of active sites and the decrease in *∆A*. Moreover, the increased layer thickness with the additional composite layer(s) results in a longer distance for electrons to transfer from active sites at the composite surface to the PB layer, leading to the reduction of *∆A*.

#### 3.5.2. Composite Sensor Response to Different •OH Radical Concentrations

**Figure 9:**
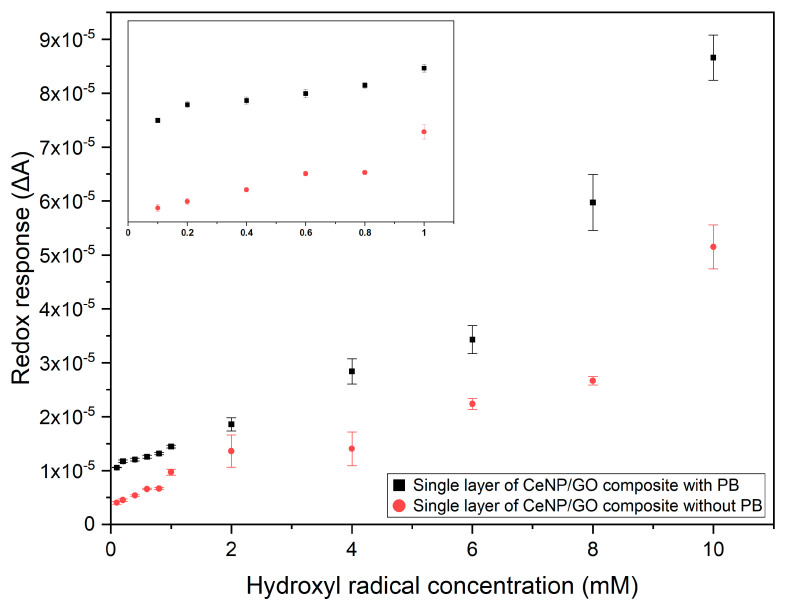
Relationships of the redox response (*∆A*) with •OH radical concentrations from 0.1 to 10 mM. The inset is for the radical range of 0.1–1.0 mM.

The single layer of composite modified sensors with and without the PB deposition were used to detect •OH radicals in the concentration range from 0.1 to 10 mM as shown in Figure 9. Both the modified composite sensors show linear relationships between the *∆A* and different concentrations of •OH radicals with R-square (R^2^) values equal to 0.93 and 0.89 for with and without the PB deposition, respectively. A higher R^2^ value of composite sensor with the PB deposition could be yielded from the electrocatalytic property of PB, which improves both conductivity and sensitivity as hypothesized before. Furthermore, the CeNP/GO composite modified sensors with the PB deposition shows a higher *∆A* for all the tested •OH radical concentrations than that without a PB layer in Figure 7 and Figure 8. The limits of detection (LOD) for the composite sensor, calculated by the equation, (3.3 × SD)/b [56], where SD and b represent the standard deviation and a slope of the regression line, are 60 and 100 µM with and without the PB modification, respectively. The electrocatalytic effect of PB is the main factor contributing to a better sensor performance in terms of *∆A* and LOD of the composite sensor. The LOD of this CeNP/GO composite sensor with the PB deposition are found to be comparable to other sensors, which are in the range of 1–100 µM [37,57,58,59].

### 3.6. Effects of PB Degradation on Sensor Performance

**Figure 10:**
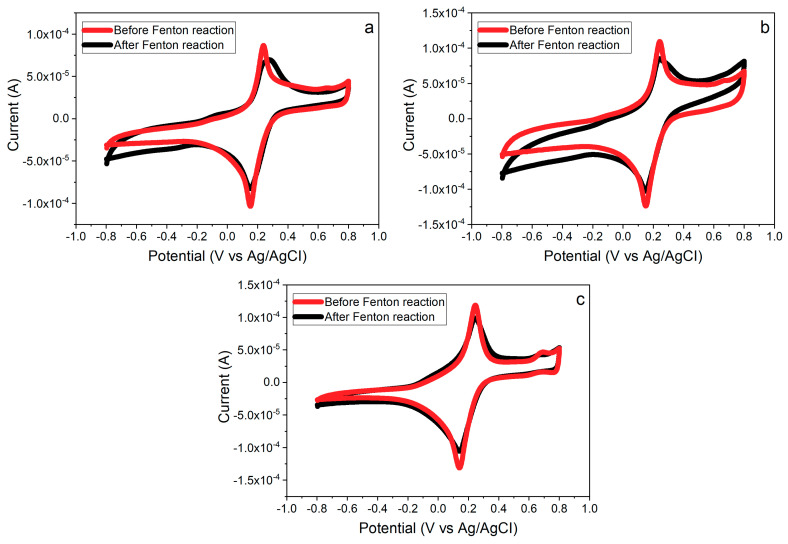
CV responses of (**a**) the single layer of CeNP/GO composite on the PB modified GCE, (**b**) double layers of CeNP/GO composite on the PB modified GCE, and (**c**) triple layers of CeNP/GO composite on the PB modified GCE after running in the Fenton reaction.

**Figure 11:**
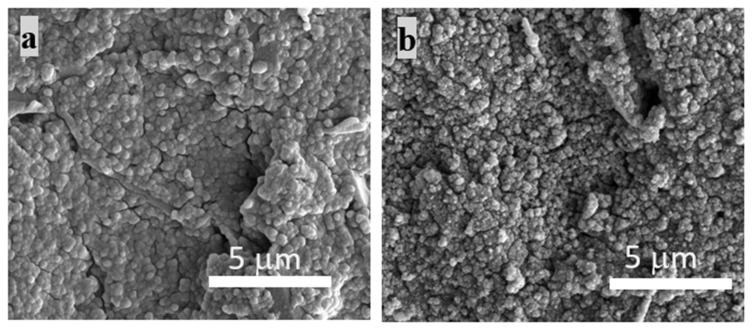
SEM images of the single layer of PB on a screen printed carbon electrode (GCE) (**a**) before and (**b**) after exposing to •OH radicals.

PB turns out to be an important layer to improve the sensor conductivity and sensitivity. As mentioned before, however, PB is found to be degraded by oxidizing species, H_2_O_2_ and •OH radicals. Since PB is used as the electrocatalyst to improve the electron transfer for redox reactions, the degradation of PB surely impacts *∆A* of this composite sensor. Cyclic voltammograms of three different composite layers with the PB deposition before and after running in the Fenton reaction are showed in Figure 10. *∆As* of all composites with single, double, and triple layers are observed to decrease after performing the detection of •OH radicals regardless of the thickness of layer. To confirm the reduction of *∆A* in Figure 10 resulting from the PB degradation, SEM images of the PB layers before and after the Fenton reaction are shown in Figure 11. Figure 11a shows the homogenous structure of PB layer, whereas a damaged rough surface of PB layer is shown after exposure to •OH radicals in the Fenton reaction in Figure 11b.

**Figure 12:**
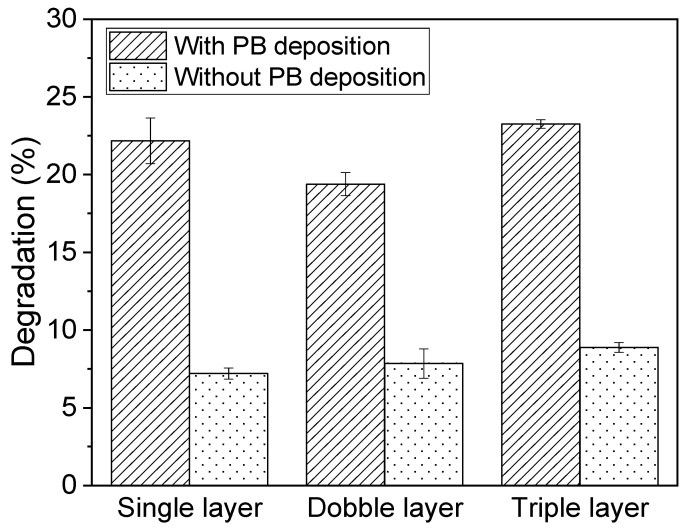
Degradation of three different composite layers on the PB modified GCE before and after running in the Fenton reaction.

In Figure 12, the percent decreases of the sensor conductivities are estimated as 22.1%, 19.4%, and 23.2% for the single, double, and triple composite sensors with the PB deposition, respectively. On the other hand, the composite sensors without the PB deposition show the 7.2%, 7.8%, and 8.8% decreases in sensor conductivity for the single, double, and triple composite layers. From Figure 12, all the composite sensors of three different layers with the PB deposition show approximately three times more degradation compared to those without the PB deposition. From the experimental results in Figure 10, Figure 11 and Figure 12, it is concluded that the decrease of *∆A* mainly results from the degradation of PB layer on the composite sensor. In addition, the different thicknesses of composite layer(s) (single, double, and triple) show no effect on the protection of PB from degradation.

## 4. Conclusions

The CeNP/GO composite deposited on the PB modified GCE is successfully synthesized by the electrochemical deposition and the drop casting method. The single layer of CeNP/GO composite sensor shows its sensitivity with •OH radicals as it produces the current increase of 87 ± 6.2 μA in CV when contacts with •OH radicals, whereas the current increases by 37 ± 0.5 μA with H_2_O_2_. The composite sensors with and without the PB modification show the linear relationships of redox response with •OH radical concentrations from 0.1 to 10 mM with the LOD as 60 and 100 µM, respectively. The PB layer is found to be a crucial factor as an electrocatalyst to improve the sensor efficiency in terms of the redox response and the LOD. Unfortunately, the PB layer is found to degrade when exposed to •OH radicals or H_2_O_2_. The double and triple composite layers show no effect on preventing the degradation of PB. Moreover, the double and triple composite layers produce lower current responses than the single composite layer. The optimum sensor configuration for •OH radical detection is the PB modified electrode with one layer of CeNP/GO composite. This work presents the promising results on the integration of PB and CeNPs to develop the electrochemical sensor for the detection of •OH radicals. Moreover, the PB degradation by •OH radicals is confirmed in this study.
